# Ten Strategies for Optimizing Ultrasound Instruction for Group Learning

**DOI:** 10.7759/cureus.1129

**Published:** 2017-04-02

**Authors:** Adaira Landry, John Eicken, Kristin Dwyer, Janet Hoyler, Trish Henwood, Sarah Frasure, Heidi Kimberly, Mike Stone

**Affiliations:** 1 Emergency Medicine, Brigham and Women's Hospital; 2 Emergency Medicine, Brown University

**Keywords:** ultrasound, education, group learning, workshop

## Abstract

Ultrasound use is rapidly increasing in clinical care and as an educational modality. While there is widespread interest in training health-care professionals to incorporate ultrasound into their daily practice, there are few resources available to guide instructors in the design of impactful and efficient training sessions. We present 10 practical strategies to optimize the educational value of ultrasound workshops for any audience.

## Editorial

### Introduction

Ultrasound is a useful diagnostic tool that is non-invasive, radiation-free, and accessible at the patient’s bedside. Both medical and surgical specialties have recognized the value of ultrasound to help with clinical diagnosis and procedural guidance [1].

As a result of the increase in demand for ultrasound education, we have noted a concomitant expansion in the range of learners, from undergraduate students to senior faculty members with decades of clinical experience. Many undergraduate programs and medical schools now incorporate ultrasound into their curricula [2]. In addition, residency programs include ultrasound instruction as part of the core curricula for residents. We are also seeing emergency departments starting to follow with requirements for attendings as well [3].

Learning ultrasound requires the integration of multiple skill sets including identification of appropriate patients, image acquisition, image interpretation, and integration of findings into the clinical management of patients. Some of these skills can be learned through lectures, web-based training, simulation, and non-traditional educational media (electronic books, podcasts). ‘Hands-on’ bedside ultrasound instruction, however, is a unique component of ultrasound education. It provides the opportunity for the learner to become facile with the ultrasound equipment, develop hand-eye coordination, gain exposure to different ultrasound exam core competencies, and practice obtaining the standard views with an instructor.

Drawing from our collective experience of educating a wide range of learners, as a group of Ultrasound Fellowship trained physicians and an ultrasound technician, we present 10 strategies to organize and teach effective group ultrasound sessions. The 10 strategies chosen were based on a consensus list we created from our personal experiences of witnessing the strengths and weaknesses of ultrasound group sessions, or workshops, as both learners and teachers. While many of these strategies can be applied to general education, we feel that ultrasound education requires a particular set of strategies that are specifically crucial to the success of ultrasound education. We believe these strategies can be applied to singular or longitudinal sessions and with both small or large groups of learners.

### Strategies

Strategy 1

Gather a needs assessment of your learners’ baseline knowledge: ultrasound workshops generally consist of learners with a variety of experiences and needs. Prior literature has also shown similarly the importance of performing a needs assessment in learners to optimize their learning [4]. In order to make the ultrasound session relevant to all learners, it is important to qualify their baseline knowledge and background as well as expectations.

One method that you can employ to determine the learner’s ultrasound knowledge is to gather a needs assessment prior to the workshop. Questions can target details regarding prior instruction (e.g., have you previously been taught the basics of ultrasound?), types of machines used (e.g., which machine types are you most familiar with?), understanding of the material you intend to teach (e.g., how comfortable are you with performing an Extended Focused Assessment with Sonography for Trauma [eFAST]?), and their expectations of the workshop (e.g., what would you like to learn during this course?). These assessments ideally are distributed to your learners prior to the workshop, providing you with enough time to adjust your curriculum accordingly.

While a formal needs assessment or survey is helpful, often we informally assess our learners at the beginning of the course itself with a few verbal questions.

Knowing your audience upfront will allow you to properly tailor the course to their level of training and personal interests. If space and time permits, consider separating participants based on their interest, level of training, and knowledge gaps. From our personal experience, instructors have an easier time teaching concepts to small groups divided by similar levels of experience. A frequent pitfall occurs when an advanced ultrasound user is paired with a learner who does not understand the basics of ultrasound. If this occurs, the advanced learner is at risk for being under stimulated while the early learner could feel behind. If unable to separate out learners by skill level, one can try having the advanced learner teach the basics to the early learner to keep both parties engaged.

Strategy 2

Focus on two or three broad teaching points: a common pitfall for an instructor or workshop leader is to teach excessive modalities in one session. A single session should not aim to cover a plethora of information. As instructors, we must not overwhelm learners; instead, we should be focused and choose our teaching points carefully.

As proficient users, it is often tempting for teachers to delve into exam specifics like measurements, advance knobology, and rarely identified pathology. Yet, such details may confuse the learner and detract him from the core goals of the session. A novice should become a master in identification, location, and documentation of relevant structures before advanced techniques are offered. Therefore, it is beneficial to identify two or three key points that you want learners to absorb by the end of the session. The information should be in a reasonable and attainable amount to be comfortably comprehended in a short window of time.

As with most skills, mastery of ultrasound is acquired over time with deliberate practice. To help aid with this we recommend "microdebriefing" after each learning point so that students can get real-time feedback and can continue to improve their skill-set as they advance in the workshop [5].

Strategy 3

Maintain a low teacher-to-student ratio to maximize educational experience: successful ultrasound instruction requires the active-sustained attention of the learners. The instructor’s goals are to teach image acquisition and image interpretation while keeping the session dynamic and entertaining.

With larger groups of learners, there can be various levels of participation and risk of disengagement. A single instructor teaching multiple learners at one machine ensures inadequate ‘hands-on’ time with the transducer and degrades the learning environment. It is up to the workshop organizer to find an adequate number of teachers to maintain a low teacher-to-student ratio, ideally 1:4 or less, in order to avoid loss of learner engagement and provide adequate ‘hands-on’ time for each participant.

One strategy we have found effective is to assign students roles during the session. Assign one learner the role of scanner; this individual will perform the scanning. Assign another learner the role of being the instructor; this person will help the scanner adjust the transducer and machine controls. Assign other learners the role of observer; they will monitor and subsequently report on the verbal communication and scanning techniques utilized during the session. Encourage the learners to switch roles over the course of the workshop.

Strategy 4

Incorporate case-based teaching and clinical correlation into your group session: learners are often taught basic technical skills with a limited understanding of how these new skills should be applied clinically. It is helpful to build in clinical relevance with these sessions, being mindful not to detract from the core two-three teaching points mentioned in Tip 2.

Case-based learning allows the educator to engage all learners simultaneously, not just the learner who is actively scanning. The ability of the teacher to reproduce both routine clinical cases and rare, yet, high acuity clinical situations, is a significant benefit provided by simulation-based ultrasound education. This approach also allows the learner to encounter abnormal or critical findings in a safe environment. Integration of ultrasound and clinical scenarios can be a useful teaching strategy for the learners.

Strategy 5

Bring images of abnormal findings to share; your models are unlikely to exhibit relevant pathology: despite the significant advantage of ‘hands-on’ bedside ultrasound sessions during workshops, ultrasound models are usually young healthy volunteers. Thus, learners are unlikely to visualize any pathology while scanning. Incorporating abnormal pathology into bedside learning sessions in the form of slides or video clips is highly educational. A common pitfall for an instructor is to only verbally describe a pathologic finding; however, it is more helpful for both the teacher and the learner if an actual video or image could be demonstrated instead. Organize the relevant abnormal findings into a slideshow prior to the session and plan to demonstrate on a large monitor or handheld mobile device when needed.

If available, simulation and task trainers can also assist in the demonstration of pathology. This is a benefit because while manipulating the ultrasound probe the learner can scan through the pathology in real time.

Strategy 6

When teaching a session focus on the core applications: introduce your students to core concepts and applications. Resist the temptation to teach sophisticated concepts until a solid framework exists. The needs assessment should dictate what your target audience will need to know. Determine what is considered a core application for your audience and teach those before teaching ancillary techniques.

For a group of emergency medicine physicians, for instance, one method that you can employ to focus on core applications is to divide ultrasound into lifesavers and time-savers. Life-saving applications include FAST (i.e. intraperitoneal free fluid in trauma), cardiac (i.e. pericardial effusion with tamponade), aorta (i.e. ruptured abdominal aortic aneurysm), obstetrics (i.e. confirming intrauterine pregnancy), lung (i.e. pneumothorax), and procedural guidance (i.e. peripheral intravenous and central lines). These core life-saving applications should have a dedicated station. Once students demonstrate competence with ‘life-saving’ applications, they may progress to ‘time-saving’ applications such as renal, biliary, deep venous thrombosis, ocular, bowel, soft tissue, skeletal, and nerve blocks. Starting with life-saving techniques before advancing to time-saving applications encourages students to focus on core concepts.

For each group of learners, you might have a different approach to what is considered fundamental and advanced. However, we have found it good practice to always have learners comfortable with core concepts before teaching more difficult or ancillary advanced techniques.

Strategy 7

Collaborate with other disciplines, as effective ultrasound teaching requires a variety of areas of expertise: ultrasound is utilized by a wide variety of medical specialties [1]. In our experience, workshops which include instructors from a variety of specialties, such as Emergency Medicine, Radiology, Cardiology, Rheumatology, and Anatomy have yielded successful group sessions.

Early and frequent collaborative efforts also serve to create a sustainable, vertical ultrasound curriculum. Through ongoing collaboration, instructors are able to expand their ultrasonographic skillset and are less likely to be placed in situations where they are uncomfortable teaching material. Furthermore, when asked questions from learners on material outside a teacher’s area of expertise, the teacher can quickly direct questions to other faculty members.

Strategy 8

Provide a suitable amount of supplies to keep the flow of the session running smoothly. Keep and enforce time limits of your group session: having adequate supplies is imperative to running a successful ‘hands-on’ ultrasound session. Listed below are critical steps for any ultrasound workshop.

a. Confirm you have enough ultrasound machines, ultrasound gel, and appropriate transducers to run the session.

b. Organize groups of educators from a variety of specialties and levels of training. Maintain a teacher-to-student ratio of 1:4 or less. One instructor should be dedicated to the timing of sessions.

c. Pillows, sheets, towels, and soft exam tables will keep your models comfortable.

d. Ideally, students enrolled in the ultrasound course should not serve as ultrasound models. A pitfall occurs when the learner serves as a model and is unable to visualize the screen while being scanned and therefore misses learning opportunities. If possible, put together list groups of volunteers (medical student or nursing student interest groups) who can serve as your ultrasound models.

Strategy 9

Have a system in place for assuring safety and comfort of models: always inform your models of what techniques will be performed during the workshop - this allows them to dress appropriately. Only those who volunteer should be chosen to act as models; no one should feel coerced into modeling. Once volunteers are chosen, groups can be formed to ensure each group has an equal number of models.

You should have a protocol to address the identification of pathology in your models (kidney cysts, gallstones, deep venous thrombosis, pregnancy, etc.). In our protocol, any incidental ultrasonographic findings result in a visit to the university health services where models see a physician and a comprehensive radiology study is performed as indicated.

Strategy 10

Allow participants to learn the material prior to arrival. Use the session to reinforce ideas and clarify knowledge gaps: ultrasound education lends itself well to the “flipped classroom” approach. In this setting, the didactic material is provided in advance, allowing workshop time to be dedicated to ‘hands-on’ scanning. Tips on how to “flip the classroom” have been published to help guide teachers through the process of curriculum transformation. If a teacher does not have personal core content to share with students, there are numerous public resources available (i.e. blogs, podcasts, videocasts, and PDFs of journal articles).

Both the learner and teacher benefit from a “flipped classroom” model. The learner will approach a ‘hands-on’ ultrasound session with more confidence and the teacher can focus on creative techniques to reinforce core content and address knowledge gaps.

When learners and teachers embrace a “flipped classroom” approach, the educational experience is both more effective and more rewarding.

### Conclusion

Ultrasound has become increasingly more integrated into the clinical practice of a wide range of medical specialties. With a growing number of learners come opportunities to advance and optimize group-based educational sessions. While not completely inclusive, these 10 strategies provide guidance to design and execute high yield ultrasound-training workshops.
